# Short chain fatty acids produced by *Cutibacterium acnes* inhibit biofilm formation by *Staphylococcus epidermidis*

**DOI:** 10.1038/s41598-020-77790-9

**Published:** 2020-12-04

**Authors:** Kouki Nakamura, Alan M. O’Neill, Michael R. Williams, Laura Cau, Teruaki Nakatsuji, Alexander R. Horswill, Richard L. Gallo

**Affiliations:** 1 Department of Dermatology, University of California San Diego, 9500 Gillman Dr. #0869, La Jolla, CA 92093 USA; 2 SILAB, R&D Department, Brive, France; 3 Department of Immunology and Microbiology, University of Colorado School of Medicine, Aurora, USA

**Keywords:** Antimicrobials, Applied microbiology, Biofilms, Microbial communities

## Abstract

Biofilm formation by bacterial pathogens is associated with numerous human diseases and can confer resistance to both antibiotics and host defenses. Many strains of *Staphylococcus epidermidis* are capable of forming biofilms and are important human pathogens. Since *S. epidermidis* coexists with abundant *Cutibacteria acnes* on healthy human skin and does not typically form a biofilm in this environment, we hypothesized that *C. acnes* may influence biofilm formation of *S. epidermidis*. Culture supernatants from *C. acnes* and other species of *Cutibacteria* inhibited *S. epidermidis* but did not inhibit biofilms by *Pseudomonas aeruginosa or Bacillus subtilis*, and inhibited biofilms by *S. aureus* to a lesser extent. Biofilm inhibitory activity exhibited chemical properties of short chain fatty acids known to be produced from *C. acnes.* The addition of the pure short chain fatty acids propionic, isobutyric or isovaleric acid to *S. epidermidis* inhibited biofilm formation and, similarly to *C. acnes* supernatant, reduced polysaccharide synthesis by *S. epidermidis*. Both short chain fatty acids and *C. acnes* culture supernatant also increased sensitivity of *S. epidermidis* to antibiotic killing under biofilm-forming conditions. These observations suggest the presence of *C. acnes* in a diverse microbial community with *S. epidermidis* can be beneficial to the host and demonstrates that short chain fatty acids may be useful to limit formation of a biofilm by *S. epidermidis*.

## Introduction

As much as 40–80% of bacteria in the terrestrial environment assemble into biofilms^1^. These biofilms provide mechanical stability and protection from the extracellular environment and can be composed of a matrix with variable polymeric substances such as polysaccharides, proteins, and extracellular DNA^2,3^. When bacterial biofilms form on foreign implanted devices, or on chronic wounds, this can result in persistent and recalcitrant infection that is more resistant to antibiotic treatment^4^. At present, limited options are available to inhibit or disrupt biofilms^5^. Therefore, there is a need to better understand mechanisms to inhibit biofilm formation and thus develop new strategies to limit their deleterious effects to human health.

Although some reports have detected some biofilm formation on healthy human skin, biofilms are not readily apparent on the skin when it is not damaged or diseased^6^. This is somewhat surprising as healthy human skin is inhabited by several bacterial genera that could potentially form a biofilm, particularly species belonging to *Staphylococcus*, *Corynebacterium*, and *Cutibacterium*^7^. Coagulase-negative Staphylococci (CoNS) such as *Staphylococcus epidermidis* and the facultative anaerobic bacterium *Cutibacterium acnes*, formerly known as *Propionibacterium acnes*, are particularly abundant on human skin^8^. Furthermore, CoNS and *C. acnes* are present at approximately 100 × density in the 5 × 10^6^ follicles present on an average adult^7^. We hypothesized that the dense bacterial population in the hair follicle would foster development of a biofilm without additional innate mechanisms in place to inhibit or disrupt biofilm formation.

In this paper, we examined if metabolites produced by *C. acnes* might limit the capacity of *S. epidermidis* to form a biofilm. Our observations show that culture supernatant from *C. acnes* can inhibit biofilm formation by *S. epidermidis.* We further demonstrate that short chain fatty acids (SCFAs), which are known metabolic products of *C. acnes*^9^, will recapitulate the action of *C. acnes* culture supernatant and can enhance susceptibility to antibiotics. These findings reveal how communication in a diverse bacterial environment can benefit the host.

## Results

### *C. acnes* inhibits *S. epidermidis* biofilm formation

**Figure 1:**
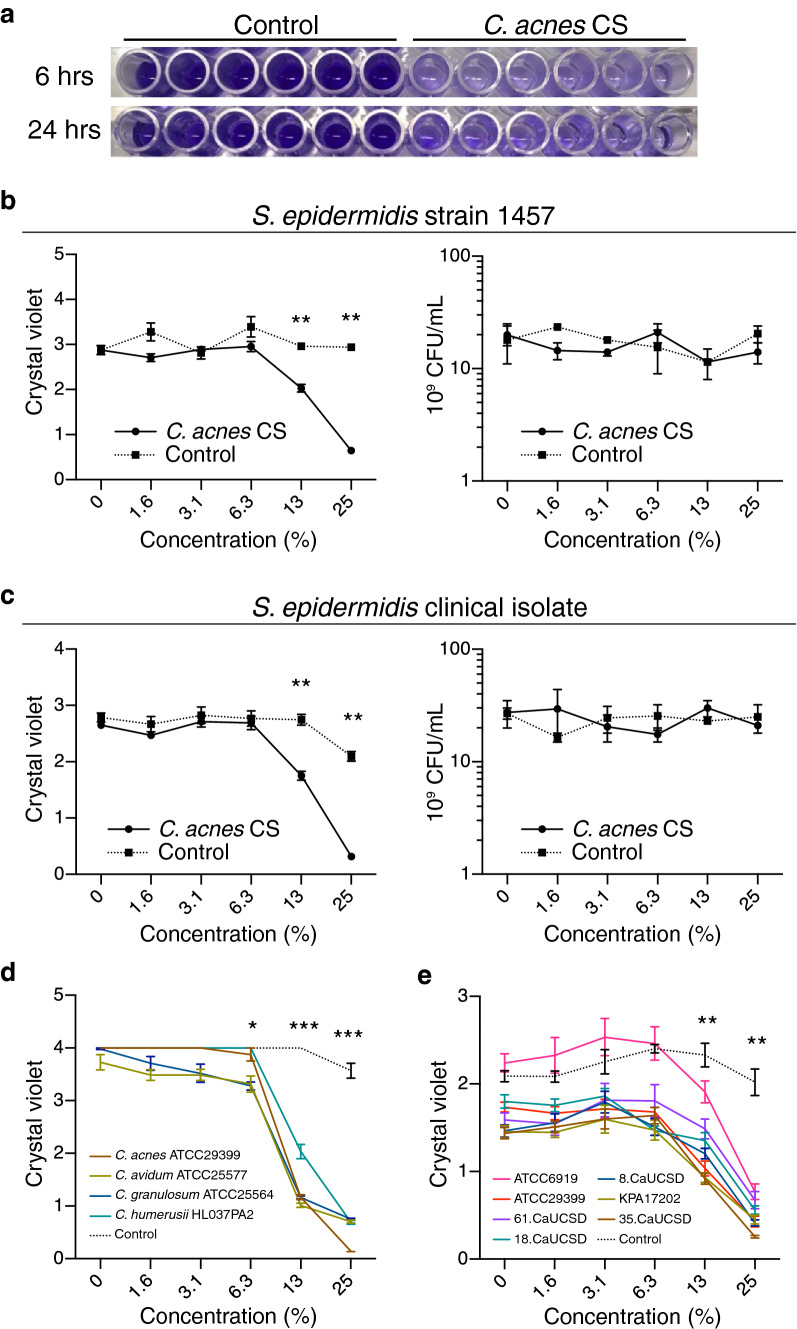
*Cutibacteria* inhibited the capacity of *S. epidermidis* to form a biofilm. (**a**) Culture supernatant (CS) of *C. acnes* ATCC29399 inhibited biofilm formation by *S. epidermidis* s 1457 as seen by crystal violet staining. *C. acnes* was cultured in reinforced clostridial media (RCM) and fresh RCM was used as control. *C. acnes* CS or RCM was added to a final concentration of 25% (v/v) during growth of *S. epidermidis* for 6 or 24 h. (**b**,**c**) Dose-dependent inhibition of biofilm formation but not cell growth by *C. acnes* CS when applied to *S. epidermidis* 1457 (**b**) or *S. epidermidis* clinical isolate (**c**). (**d**) CS of several species of *Cutibacteria *inhibited *S. epidermidis* 1457 biofilm formation. (**e**) CS of several strains of *C. acnes* inhibited *S. epidermidis* 1457 biofilm formation. Data were expressed as mean ± SEM of a single experiment (n = 6) that was representative of 3 independent experiments. Differences were analyzed using the unpaired Student’s *t* test (**b**,**c**) or one-way ANOVA with Dunnett's test (**d**,**e**). Significance was shown as *P < 0.05, **P < 0.01, *** *P* < 0.001.

*C. acnes* and *S. epidermidis* co-exist on healthy human skin and are each abundant members of the human skin microbiome^10^. *S. epidermidis* 1457 is a ST86 strain originally isolated from catheter related bacteremia and can form robust biofilms in culture^11,12^. To investigate if biofilm formation by *S. epidermidis* 1457 could be influenced by the presence of *C. acnes*, we prepared sterile-filtered culture supernatant (CS) from the anaerobic culture of *C. acnes* ATCC29399 and added this at various concentrations to *S. epidermidis* 1457. A dose-dependent inhibition of biofilm formation was observed after the addition of *C. acnes* CS (Fig. 1A). This inhibition of biofilm formation occurred without inhibition of bacterial growth up to a concentration of 25% of CS (Fig. 1B). Biofilm formation by a clinical isolate of *S. epidermidis* from healthy human skin was also inhibited after exposure to *C. acnes* CS (Fig. 1C). Inhibition of *S. epidermidis* 1457 biofilm formation was also observed following the addition of CS from other *Cutibacterium* species (Fig. 1D)*.*

*C. acnes* strains are genetically categorized to several subgroups (IA1, IA2, IB, IC, II, and III). We tested ATCC6919, ATCC29399, 8.CaUCSD, 18.CaUCSD, and 61.CaUCSD (group 1A1); KPA17202 (group 1B); and 35.CaUCSD (group II). Four of those are clinical isolates from acne lesional skin (18.CaUCSD), acne non-lesional skin (8.CaUCSD and 61.CaUCSD), and healthy skin (35CaUCSD). Each of these other strains of *C. acnes* also inhibited formation of biofilm by *S. epidermidis* 1457 (Fig. 1E). Importantly, addition of *C. acnes* CS after the formation of a biofilm by *S. epidermidis* did not disrupt the pre-existing biofilm (Fig. S1). These observations suggest that a metabolic product or products by *C. acnes* and related species inhibit biofilm formation by *S. epidermidis*. On the other hand, biofilm formation by *Pseudomonas aeruginosa* and *Bacillus subtilis* were not inhibited by *C. acnes* CS and biofilm formation by *S. aureus* was only slightly inhibited (Fig. S2A–S2C).

### Chemical properties of the *C. acnes* metabolites that inhibit *S. epidermidis* biofilm formation

**Figure 2:**
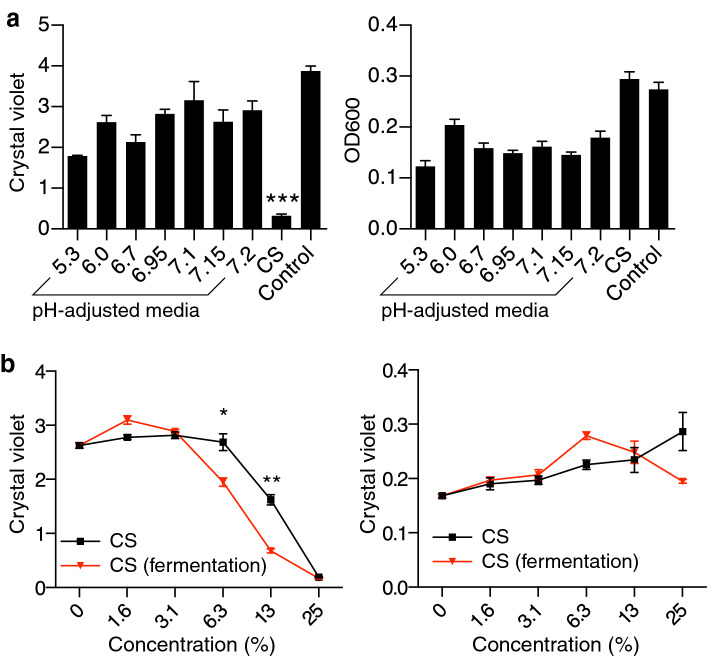
*S. epidermidis* biofilm formation is not observed at low pH but is increased during fermentation of *C. acnes*. (**a**) *S. epidermidis* 1457 was grown for 6 h in TSB culture medium at the indicated pH range following the addition of hydrogen chloride or sodium hydroxide. Biofilm formation compared to cell growth after the addition of *C. acnes* ATCC29399 CS or RCM as control were shown. Data are expressed as mean ± SEM of a single experiment (n = 6) that is representative of 3 independent experiments. Differences were analyzed using one-way ANOVA with Dunnett's test. (**b**) *C. acnes* ATCC29399 was cultured under anaerobic conditions with the addition of glycerol as a carbon source for fermentation. Sterile media from these cultures was then added at the indicated final concentrations to *S*. *epidermidis* 1457 culture. Biofilm formation assayed at 6 h was compared to the results with *C. acnes* ATCC29399 CS. Data are expressed as mean ± SEM of a single experiment (n = 6) that is representative of 3 independent experiments. Differences were analyzed using the unpaired Student’s *t* test. Significance was shown as *P < 0.05, **P < 0.01, ***P < 0.01.

To identify metabolic products of *C. acnes* that can inhibit *S. epidermidis* biofilm formation we examined the chemical properties of CS from *C. acnes* ATCC29399. To exclude the possibility that the low pH of *C. acnes* CS was responsible for inhibition of the biofilm, we measured the media pH after the addition of *C. acnes* CS (Table 1A). 25% *C. acnes* CS acidified tryptic soy broth (TSB) medium from a pH of 7.2 to a pH of 6.0. However, acidification of TSB medium to a pH of 6.0 by the addition of hydrochloric acid did not inhibit *S. epidermidis* 1457 biofilm formation or cell growth (Fig. 2A). Thus, media pH reduction by *C. acnes* was not responsible for inhibition of *S. epidermidis* biofilm production.

**Table 1:** pH analysis of *C. acnes* CS (A), and chemical properties of biofilm inhibiting activity from *C. acnes* (B).

**(A)**								
% *C. acnes* CS	100	50	25	12.5	6.3	3.1	1.6	0
pH	4.85	5.3	6.0	6.7	6.95	7.1	7.15	7.2
% RCM	100	50	25	12.5	12.5	3.1	1.6	0
pH	5.5	6.55	6.85	7.0	7.1	7.15	7.2	7.2

**(B)**								
Manipulation	Biofilm inhibition	Suggested chemical property						
Ammonium sulfate precipitate	Not in precipitate	Not protein						
Heating at 100 °C for 10 min	Remains active	Heat resistant						
Proteinase digestion	Remains active	Not protein						
Lysozyme digestion	Remains active	Not glycoprotein						
Lyophilization	Activity lost	Volatile						
MW = 500 dialysis	Activity lost	MW < 500						

*CS* culture supernatant; *RCM* reinforced clostridial media; *MW* molecular weight.

Stability analysis of the biofilm inhibitory activity produced by *C. acnes* further defined the chemical nature of the molecule(s) in the *C. acnes* CS with activity to inhibit biofilm (Table 1B). The inhibitory activity could not be precipitated from CS by the addition of ammonium sulfate and was resistant to inactivation by digestion with proteinase K or lysozyme. This suggested the bioactive compound(s) were not proteinaceous. Biofilm activity was resistant to heating in a sealed tube at 100 °C for 10 min but was lost when CS was lyophilized. Additionally, the biofilm inhibitory activity was retained after passage through a 500 Da MW filter. These results indicated that the bioactive molecule(s) produced by *C. acnes* were heat stable and volatile.

*C. acnes* is a facultative anaerobe that produces short chain fatty acids (SCFAs) when provided a carbon source such as glycerol^13^. These SCFAs are volatile, heat stable and resistant to proteases and thus matched well with the chemical properties of the biofilm-inhibiting activity in *C. acnes* CS. To determine if SCFA production by *C. acnes* correlated with inhibitory activity, we investigated if the addition of glycerol to *C. acnes* culture media increased the production of SCFAs. Indeed, CS of *C. acnes* grown in the presence of glycerol has greater potency for biofilm inhibitory activity compared to CS without glycerol supplementation (Fig. 2B). This observation further implied that SCFAs may inhibit *S. epidermidis* biofilm activity.

### SCFAs inhibit biofilm formation by *S. epidermidis*

**Figure 3:**
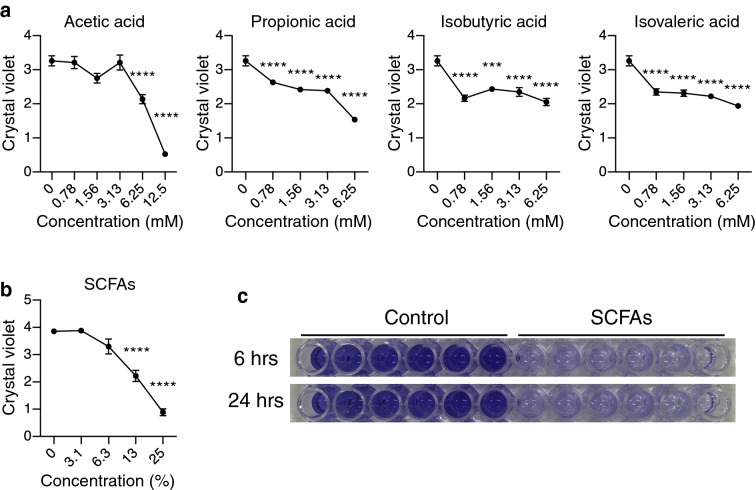
SCFAs inhibit biofilm formation by *S. epidermidis*. (**a**) SCFAs as indicated were added to culture media of *S. epidermidis* 1457 at concentrations that did not inhibit cell growth. Biofilm formation at 6 h was assayed by crystal violet staining. (**b**) A mixture of SCFAs simulating the composition measured in *C. acnes* CS inhibited biofilm formation of *S. epidermidis* 1457. (**c**) Representative images of biofilm inhibition by SCFAs were shown. SCFAs or distilled water as a control were added to a final concentration of 25% (v/v) during growth of *S. epidermidis* for 6 or 24 h. Data were expressed as mean ± SEM of a single experiment (n = 6) that was representative of 3 independent experiments. Differences were analyzed using one-way ANOVA with Dunnett's test. Significance was shown as *P < 0.05, **P < 0.01, *** *P* < 0.001, **** *P* < 0.0001.

SCFAs known to be produced by *C. acnes* include acetic acid, propionic acid, isobutyric acid, and isovaleric acid^13^. Therefore, to directly test the hypothesis that SCFAs can inhibit *S. epidermidis* biofilm, we added these pure SCFAs to *S. epidermidis* 1457 cultures. Similar to *C. acnes* CS, SCFAs inhibited biofilm formation at concentrations that did not inhibit cell growth (Fig. 3A). Of note, this inhibition occurred at physiologic concentrations of SCFAs produced by *C. acnes* on skin^13^, and was weakest for acetic acid, a SCFA produced by *S. epidermidis* as a metabolic byproduct. Furthermore, a mixture of SCFAs that mimicked the composition of SCFAs in *C. acnes* CS (acetic acid, 3.17 mM; propionic acid, 4.59 mM; isobutyric acid, 0.11 mM; isovaleric acid, 2.06 mM) strongly inhibited biofilm formation (Fig. 3B,C). These observations suggested the production of SCFAs by *C. acnes* inhibits the capacity of *S. epidermidis* to produce a biofilm.

### *C. acnes* and SCFAs increase capacity of ampicillin and doxycycline to kill *S. epidermidis*

**Figure 4:**
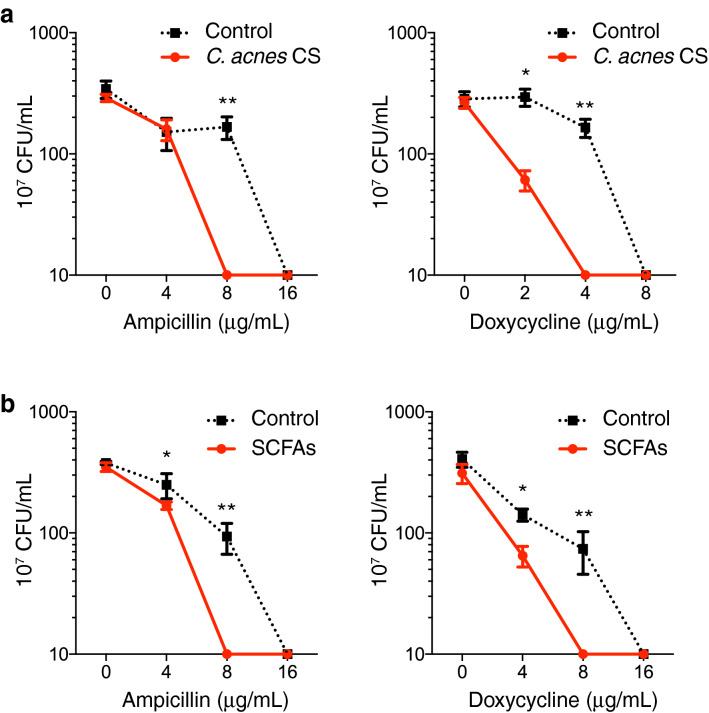
*C. acnes* and SCFAs increase sensitivity of *S. epidermidis* to ampicillin and doxycycline in killing (**a**,**b**) Minimal inhibitory concentrations of ampicillin and doxycycline were determined in the presence of *C. acnes* CS (**a**) or SCFAs (**b**). A mixture of SCFAs simulated the composition measured in *C. acnes* CS. *S. epidermidis* in TSB at 1 × 10^7^ CFU/mL was cultured for 6 h with several concentrations of antibiotics. After the incubation total CFU was counted. Data were expressed as mean ± SEM of a single experiment (n = 3) that was representative of 3 independent experiments. Differences were analyzed using the unpaired Student’s *t* test. Significance was shown as *P < 0.05, **P < 0.01.

Since biofilm formation is associated with resistance to killing by antibiotics, we tested whether *C. acnes* CS would enable antibiotics to kill bacteria grown under conditions that would otherwise lead to formation of a biofilm. *S. epidermidis* 1457 was cultured with *C. acnes* CS and with increasing concentrations of ampicillin or doxycycline. After incubation for 6 h, *S. epidermidis* was killed at lower concentrations of ampicillin or doxycycline when grown with *C. acnes* CS compared to culture medium that was not conditioned (RCM) as a control (Fig. 4A). The same result was obtained with SCFAs (Fig. 4B). This observation suggested that by inhibiting the biofilm formation, *C. acnes* or pure SCFAs can increase *S. epidermidis* susceptibility to antibiotics.

### *C. acnes* and SCFAs inhibit polysaccharide-dependent biofilm formation by *S. epidermidis*

The process of biofilm formation has at least two distinct phases: initial attachment by surface proteins and biofilm accumulation. The second phase requires cell-to-cell interaction that is mediated by intercellular polysaccharide adhesin (PIA aka PNAG). In the majority of *S. epidermidis* strains^14^, including *S. epidermidis* strain 1457, the production of polysaccharide is important for accumulation of the biofilm.

**Figure 5:**
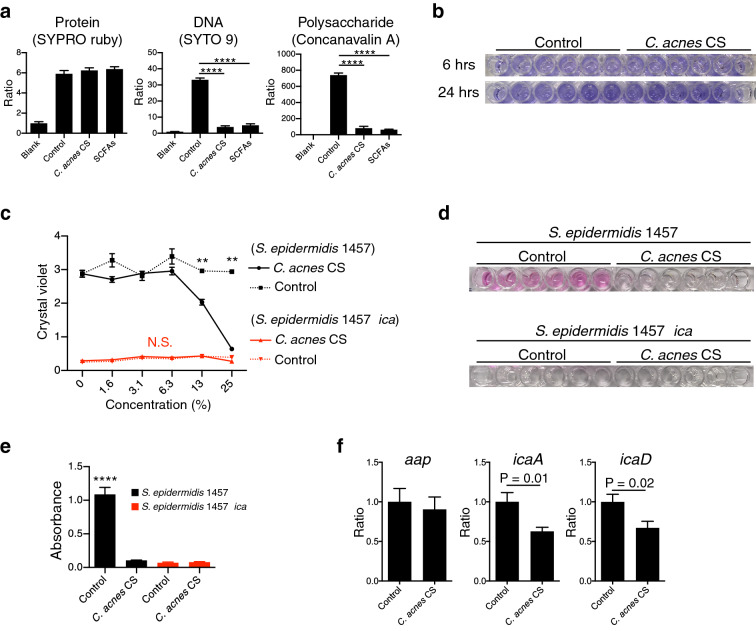
*C. acnes* CS and SCFAs inhibit assembly of polysaccharide in *S. epidermidis* biofilms. (**a**) Protein, DNA, and polysaccharides were detected by fluorescent dyes as indicated. *S. epidermidis* strain 1457 was cultured on glass plates for indicated time and stained with for each biofilm component and results of fluorometry were summarized. Differences were analyzed one-way ANOVA with Dunnett's test. (**b**) *C. acnes* CS was added to a final concentration of 25% (v/v) during growth of *S. epidermidis* strain 1457 and its Δ*ica* mutant strain for 6 or 24 h. The amount of biofilm was detected by crystal violet staining. (**c**) Dose-dependent inhibition of biofilm formation by *C. acnes* CS when applied to *S. epidermidis* 1457 or *S. epidermidis* 1457 Δ*ica* mutant strains. (**d**) Periodic acid Schiff stain of the biofilm. *S. epidermidis* strain 1457 and its Δ*ica* mutant were cultured for 6 h and then stained. (**e**) Periodic acid Schiff stain was quantified with absorbance at 550 nm. (**f**) Gene expressions of *aap*, *icaA*, and *icaD* were assayed by qRT-PCR using the *S. epidermidis* 1457 biofilm samples treated with *C. acnes* CS or control for 6 h. Differences were analyzed using the unpaired Student’s *t* test. Data were expressed as mean ± SEM of a single experiment (n = 6) that was representative of 3 independent experiments. Significance was shown as **P < 0.01, ****P < 0.0001.

To determine if *C. acnes* acts at the phase of attachment or polysaccharide assembly, we stained culture plates during formation of the biofilm with probes to detect total protein, DNA or carbohydrate. At 2 h, protein deposition by *S. epidermidis* 1457 was unchanged by *C. acnes* or SCFAs, thus suggesting no effect on initial attachment (Fig. 5A). However, after 6 h, the amount of polysaccharide and DNA was markedly reduced (Fig. 5A). Considering that SYTO 9 stains both intracellular DNA and extracellular DNA, the reduction of SYTO 9 staining can be also interpreted as a reduction of adherent bacteria.

In addition, to directly test the effects of *C. acnes* CS and SCFAs on cell adhesion compared to assembly of the biofilm, we tested the Δ*ica* mutant strain of *S. epidermidis* 1457 which lacks the production of PIA but can still assemble a protein-based biofilm^15^. As expected, biofilm formation capacity by the *S. epidermidis* 1457 Δ*ica* mutant was less than in the wild-type strain. This biofilm formed by the Δ*ica* mutant was not further inhibited by *C. acnes* CS (Fig. 5B,C), thus demonstrating that the effect of *C. acnes* does not occur in absence of polysaccharide deposition. To further confirm the inhibition of polysaccharide production, we stained biofilm with periodic acid–Schiff (PAS). PAS is a staining method used to detect polysaccharides such as glycogen, and mucosubstances such as glycoproteins, glycolipids and mucins. As expected, *C. acnes* CS inhibited the production of PAS-positive substance that was also undetectable in biofilms by *S. epidermidis* 1457 Δ*ica* mutant (Fig. 5D,E). Consistent with this observation, the expression of *icaA* and *icaD*, two major genes involved in the synthesis of intercellular adhesin by *S. epidermidis*^16^, were significantly decreased in the presence of *C. acnes* CS. In contrast, the expression of accumulation-associated protein (Aap) was unchanged (Fig. 5F), which is consistent with our observation that the *S. epidermidis* Δ*ica* mutant biofilm is unaffected by *C. acnes* CS (Fig. 5A,B). Taken together, these results suggest that *C. acnes* CS may act directly or indirectly to inhibit the synthesis or assembly of polysaccharide in the biofilm, potentially through suppression of *icaA* and *icaD* expression.

## Discussion

*C. acnes* is one of the most abundant commensals on human skin^7,17^. Other commensal skin commensal organisms such as specific strains of CoNS can kill *S. aureus*^18,19^ or selectively inhibit the growth of *C. acnes*^20^ but limited information has been found to suggest that *C. acnes* can benefit its host. In contrast, although typically present without deleterious effect, *C. acnes* can cause infection of implanted medical devices^21^, and is most frequently thought of due to its involvement in the pathogenesis of acne vulgaris^22,23^. This study sought to determine if *C. acnes* could benefit it’s host by influencing the function of *S. epidermidis* to form a biofilm*.* We conclude that short chain fatty acids produced by *C. acnes* will limit biofilm formation by *S. epidermidis.* This observation may explain in part why highly abundant and dense growth of *S. epidermidis* in the human hair follicle does not typically result in formation of a biofilm.

To test the capacity of *S. epidermidis* to form a biofilm, we examined defined laboratory strains of *S. epidermidis* isolated from infection and clinical isolates obtained from healthy skin. Similarly, we examined multiple strains of *C. acnes* as well as other related bacterial species relevant to this issue. Our initial reference strain of *C. acnes* inhibited *S. epidermidis* isolated from health and disease equally well. Importantly, this occurred at concentrations of *C. acnes* supernatant that did not inhibit the growth of the opposing species and acted only before the biofilm was formed. Other strains of *C. acnes* as well as other major members of the *Cutibacterium* genus also prevented *S. epidermidis* from forming a biofilm. This suggests the activity produced from *C. acnes* is likely conserved across the genus*.* Furthermore, the action against biofilm formation was selective. *C. acnes* CS strongly inhibited biofilms by *S. epidermidis*, inhibited biofilms by *S. epidermidis* to a lesser extent, and did not inhibit biofilms by *P. aeruginosa* or *B. subtilis*. Although these findings cannot exclude the potential that some *S. epidermidis* strains could be resistant, or that some *C. acnes* may be inactive, our observations support a general conclusion that *C. acnes* can inhibit *S. epidermidis* biofilm formation.

To determine the mechanism of biofilm inhibition, we considered the possibility that simple acidification of the environment by the *C. acnes* CS could be the source of activity. The pH at the surface of the skin is normally acidic, ranging in pH values of 4–6^24,25^. Many bacterial species, including *S. epidermidis,* also can produce substances that change the pH of the environment^26,27^, and lower pH has been associated with increased biofilm formation, not a decrease^28^. Thus, we considered it unlikely that low pH would be the mechanism of inhibition. Analysis of *C. acnes* culture medium showed a drop in pH from 5.5 to 4.85 after 14 days of anaerobic culture, and 25% mixture of *C. acnes* CS with TSB had a pH of 6.0. Since acidification of *S. epidermidis* media from 7.2 to 5.3 did not affect the formation of biofilm in our system, we conclude acidic pH is not a responsible for our observations.

We considered the possibility that *C. acnes* may produce a specific protein or peptide with the capacity to inhibit biofilm formation. Stability analysis of the biofilm inhibitory activity produced by *C. acnes* suggested this was likely not the case since the activity was volatile, protease resistant and heat resistant. Since prior reports have shown *Cutibacteria* can produce SCFAs^29^ and some SCFAs have similar chemical properties to the observed bioactivity from *C. acnes*^30^, we tested if pure SCFAs could have an effect similar to *C. acnes* CS. These experiments showed direct addition of SCFAs had a similar action to *C. acnes* CS. Although these results do not rule out the potential that other bioactive *C. acnes* metabolic products, the totality of our observations strongly support the hypothesis that SCFAs may be at least part of the explanation for how *C. acnes* acts against *S. epidermidis* biofilms. Further work to define these as the cause, and understand the mechanism of action against *S. epidermidis*, is still needed.

Our results indicated that *C. acnes* CS did not inhibit the growth of *S. aureus* but prior studies have shown that SCFAs did inhibit *S. aureus* at concentrations over 250 mM^31^. The discrepancy with our results may therefore be due to the lower concentrations of pure SCFAs present in *C. acnes* CS and which we used in these experiments. However, other molecules in the complex *C. acnes* CS mixture may also influence our observations. For example, some *C. acnes* phylogroups encode biosynthesis genes for a thiopeptide with possible antimicrobial activity against *S. epidermidis*, which conversely secretes bacteriocins such as epidermin that kills *C. acnes*^32^. The antagonism between *S. epidermidis* and *C. acnes* is also noted in acne vulgaris, in which not only SCFAs but many factors like antimicrobial peptides secreted from keratinocytes have an impact^33^. Considering that *S. epidermidis* also produces SCFAs, further study of additional, unidentified factors other than SCFAs should be addressed in the future. One example is *N*-acetylcysteine, which inhibits the growth, adhesion, and biofilm formation of Gram-positive skin bacteria^34^.

One of the clues to understanding a mechanism of action for *C. acnes* to inhibit biofilm formation was the observation that pure SCFAs that are produced by *C. acnes* had a similar effect to *C. acnes* conditioned medium. SCFAs may have multiple beneficial effects and have been studied in the setting of the intestinal microbiome^35^ and contribute to the reduction of luminal pH which could inhibit pathogenic microorganisms in gut^36^. SCFAs also can have direct antimicrobial activity^37,38^, can increase mucin production^39^, influence immune responses^40^ and suppress calcium phosphate-induced itching through activation of IL-6/p-ERK signaling^41^. In the context of the present study we also observed that higher concentrations of SCFAs can inhibit *S. epidermidis* survival. Our observations add to this list and suggest that the production of SCFAs may activate host defense, inhibit bacterial survival or act to limit biofilm production. As these effects are dose dependent they will be influenced by the environment since hypoxic conditions within the follicle will favor greater production of SCFAs. Further study is needed to determine if activity observed from *C. acnes* is solely due to SCFAs, as well as the most relevant functions of SCFA in different specific contexts seen in epithelial biology.

The bacterial biofilm matrix is mainly composed of polysaccharides, proteins, nucleic acids and lipids^42^. Since *S. epidermidis* 1457 produces a significant amount of PIA-dependent biofilm, it is considered as an excellent model strain to understand *icaADBC* transcriptional regulation^43^. Regulation of biofilm formation may vary depending on the type of biofilm produced as well as the species of organism that produces the biofilm. A previous report suggested *C. acnes* could induce *S. aureus* biofilm formation by producing coproporphyrin III^44^. *S. epidermidis* was also reported to inhibit *S. aureus* biofilm formation and nasal colonization^45^. Our observations did not find lower biofilm formation with *S. aureus* as we did with *S. epidermidis*. These vastly different responses from two somewhat similar species of Staphylococci suggest that the mechanisms by which the products of *C. acnes* act on *S. epidermidis* are specific. We hypothesize that polysaccharide synthesis or assembly is a primary target for SCFAs and *C. acnes* CS and we are working to define this mechanism of action. A series of experiments supported this idea. Staining with SYTO 9 showed less staining (intracellular DNA and extracellular DNA) when SCFAs are added, but bacterial growth itself was not inhibited by these concentrations of SCFAs. This suggests that less bacteria were able to adhere and form a biofilm in the presence of SCFA rather than a decrease in DNA synthesis.

While the impact of SCFAs on epithelia is being gradually elucidated, little is known about how SCFAs interact with other microbes on skin. Our data add here a new level of insight and suggest that production of SCFAs by *C. acnes* is an important mechanism to maintain homeostasis of the microbiome in the cutaneous environment. This may be particularly important in the approximately 5 × 10^6^ follicles present on adult human skin where the density of *S. epidermidis* is high and hair shafts are present. Such an environment might be expected to foster the frequent development of a biofilm. Despite high density colonization by *S. epidermidis*, biofilms rarely appear on healthy intact skin. We speculate the observations reported here may be one of the factors that limits biofilm formation and enables homeostasis between *S. epidermidis* and the host environment. Understanding of mechanisms to maintain the normal balance between humans and commensal microbes may be applicable for development of new strategies to prevent biofilm formation in wounds and medical devices.

## Methods

### Experimental design

This study was designed to biochemically characterize the activity of *C. acnes* inhibition of *S. epidermis* biofilm formation. Pilot experiments were performed to determine the activity. Experimental replicates of at least three (indicated in figure legends) were performed and analyzed to determine statistical significance as defined by *P* < 0.05. Sample analysis was performed quantitatively in an unblinded manner and confirmed by at least three independent experiments as indicated in the figure legends.

### Bacterial culture

Preparation of bacterial cultures was performed as follows. Bacterial stocks frozen at − 80 °C in TSB (Sigma-Aldrich, St. Louis, MO) with 20% glycerol was inoculated into 5 mL of TSB. The culture was aerated by shaking at 120 rpm at 37 °C and grown overnight. Proper concentration of antibiotics was added if bacteria strain contains resistance genes for positive selection.

### Crystal violet assay for biofilm formation

*S. epidermidis* 1457^12^, *S. epidermidis* clinical isolate, *S. aureus* USA300^46^, *S. aureus* RN4220^47^, *P. aeruginosa* PAO1^48^, *P. aeruginosa* P4^49^, and *B. subtilis* strain ATCC6051 were inoculated into 3% TSB medium, and cultured at 37 °C overnight. Then, the culture was diluted in fresh TSB medium to 1 × 10^7^ CFU/mL by 600-nm optical density. A total of 100 mL of each diluted culture was transferred to flat-bottom 96-well microtiter polystyrene plates (Fisher Scientific, Waltham, MA). The plates were then incubated for 6 h or 24 h at 37 °C without shaking. After 6 h or 24 h of incubation, the supernatants were removed by washing the plates three times using 200 mL of normal saline. Subsequently, 100 mL of 0.01% crystal violet (CV) solution was added to all wells containing completely dry biofilm. After 15 min of dyeing, the excess CV was removed by washing twice with sterile water. Eventually, the fixed CV was released by 33% acetic acid and the absorbance detection at 595 nm was measured^50^.

### Preparation of *Cutibacterium* culture supernatant

All *Cutibacteria* species, including all the *C. acnes* strains, were cultured in RCM media (Sigma-Aldrich, St. Louis, MO), anaerobically for 14 days^13^. Culture media was then centrifuged for 10 min and this media was then filtered through a 0.22 micron filter (Fisher Scientific, Waltham, MA) to produce culture supernatant (CS). In some experiments ammonium sulfate was added to *C. acnes* CS, and the solution was centrifuged at 10,000* g* for 10 min. At the concentration of 60%, 70%, and 80% (w/v) of ammonium sulfate, precipitate was confirmed. The precipitate was collected and used for further analysis of anti-biofilm activity. *C. acnes* CS was also tested by lyophilization using SpeedVac Vacuum Concentrators (Thermo Fisher Scientific, Waltham, MA). Volatile portion of a sample was removed by evaporation. For dialysis, *C. acnes* CS was centrifuged with cellulose membrane (Amicon Ultra Centrifugal Filters; Millipore Sigma, Burlington, MA) to determine the rough molecular weight of the activity. After confirming that the molecular weight was under 3,000 Da, flow-through from the column was set to the dialysis tubes (Float-A-Lyzer Dialysis Devices; Spectrum Chemical Manufacturing, New Brunswick, NJ), and dialyzed in a clean floating water for 24 h. The concentration of SCFAs produced by laboratory strains of *C. acnes* strain ATCC29399 was measured as previously determined^13^. Briefly, bacteria were cultured under anaerobic conditions for 14 days. SCFAs concentrations in culture supernatants were measured by gas chromatography–mass spectrometry after ethyl acetate extraction. Concentrations were as follows: acetic acid, 3.17 mM; propionic acid, 4.59 mM; isobutyric acid, 0.11 mM; isovaleric acid, 2.06 mM. All SCFAs were purchased from Sigma-Aldrich (St. Louis, MO).

### Colony forming assay

*S. epidermidis* was inoculated into 3% TSB medium, and cultured at 37 °C overnight. Then, the culture was diluted in fresh TSB with 25% of *C. acnes* CS or RCM to 1 × 10^7^ CFU/mL by 600-nm optical density. Ampicillin sodium salt (Sigma-Aldrich, St. Louis, MO) or doxycycline hyclate (Sigma-Aldrich, St. Louis, MO) with several final concentrations were also added. A total of 100 μL of each diluted culture were transferred to flat-bottom 96-well microtiter polystyrene plates in which a 5 mm plastic cover slip coupon was put inside. The plates were then incubated for 6 h at 37 °C without shaking. A coverslip was collected from the plates, and we extracted bacteria in biofilm using vortex mixer and sonication^51^. Colony forming unit was counted on trypticase soy agar plate.

### Fluorescent staining of biofilms

Major components of the biofilm (protein, DNA, and polysaccharide) were visualized by fluorescent dyes. Protein was detected with FilmTracer SYPRO Ruby Biofilm Matrix Stain (Thermo Fisher Scientific, Waltham, MA), and observed under microscopy at red channel. DNA was detected with SYTO 9 Green Fluorescent Nucleic Acid Stain (Thermo Fisher Scientific, Waltham, MA), and observed under microscopy at green channel. Note that both intracellular DNA and extracellular DNA are stained with SYTO 9. Polysaccharide was detected with Concanavalin A, Alexa Fluor 350 Conjugate (Thermo Fisher Scientific, Waltham, MA), and observed under microscopy at blue channel. Staining was quantified using a fluorometer. Excitation/emission wavelengths were 450 nm /610 nm for SYPRO Ruby, 480 nm/500 nm for SYTO 9 Green, and 346 nm /442 nm for Concanavalin A, Alexa Fluor 350 Conjugate, respectively.

### Periodic acid-Schiff colorimetric assay

Periodic Acid Schiff (PAS) Stain Kit (ab150680; Abcam, Cambridge, MA) was used to detect polysaccharide. The methods to quantify in a microtiter plate format is described elsewhere^52^. Briefly, after the formation of bacteria, 100 μL of periodic acid was added to the plate and incubated for 30 min. After the washing, 100 μL of Schiff’s reagent was added and incubated for 15 min. Absorbance was measured at 550 nm in a plate reader.

### DNA/RNA purification, reverse transcription, and quantitative real-time polymerase chain reaction (qRT-PCR)

Bacterial DNA and RNA were purified using ZymoBIOMICS DNA/RNA Miniprep Kit (Zymo Research, Orange, CA). Total RNA from each sample was reverse-transcribed into cDNA using the iScript cDNA synthesis kit (Bio-Rad, Hercules, CA). Gene expression levels were determined by quantitative real-time reverse transcription PCR using iTaq Universal SYBR Green Supermix (Bio-Rad, Hercules, CA) in triplicates. mRNA levels of target genes were normalized to those of the 16S rRNA gene by the 2^−ΔΔCt^ method. The primer sequences for target genes were as follows: *aap*, forward 5′-TGATCGGATCTCCATCAACT-3′ and reverse 5′-AAGGTAGCCAAGAGGACGTT-3′; *icaA*, forward 5′-CTCTTGCAGGAGCAATCAAT-3′ and reverse 5′-AGAGCACGTGGTTCGTACTT-3′; *icaD*, forward 5′-GAGGCAATATCCAACGGTAA-3′ and reverse 5′-AAATTTCCGTGTTTTCAACATT-3′. The sequences of the universal 16S rRNA primers (V1–V3 region) was as follows: forward 5′-AGTGAAAGACGGTCTTGCTGTC-3′ and reverse 5′-ATTGCGGAAGATTCCCTACTG-3'.

### Statistics

Statistical analysis was performed with Prism software (version 6; GraphPad Software). Results are expressed as mean ± SEM. *P* values less than 0.05 were considered significant.

## Supplementary information

Supplementary figure legends.

Supplementary figures.

## Data Availability

No data sets were generated or analyzed in this study.
