# Supplementary material for: Short chain fatty acids produced by Cutibacterium acnes inhibit biofilm formation by Staphylococcus epidermidis
Source: Sci Rep. 2020 Dec 4;10:21237. doi: 10.1038/s41598-020-77790-9 (PMC7718897; doi:10.1038/s41598-020-77790-9)
Supplement: Supplementary file 1 — Supplementary figure legends. [file 41598_2020_77790_MOESM1_ESM.docx]

**FIG S1. *C. acnes* ATCC29399 CS did not disrupt the pre-existing biofilm**

Biofilm of *S. epidermidis* 1457 was formed on a plastic plate by culturing for 24 hours. After the plate was washed, indicated concentrations of *C. acnes* ATCC29399 CS was poured on the biofilm and incubated for 3 hours or 6 hours. The remaining biofilm was stained by crystal violet. Data are expressed as mean ± SEM of a single experiment (n = 6) that is representative of 3 independent experiments. Differences were analyzed one-way ANOVA with Dunnett's test.

**FIG S2. *C. acnes* ATCC29399 inhibited biofilm formation of  *S. aureus* USA300 and *S. aureus* RN4220 at a high concentrations, but did not inhibit biofilm formation by *P. aeruginosa* PAO1, *P. aeruginosa* P4, and *B. subtilis* strain ATCC6051**

Biofilm formation and cell growth during exposure to *C. acnes* ATCC29399 CS or RCM as control was assayed. Two *S. aureus* strains (USA300 and RN4220, **a**), two *P. aeruginosa* strains (PAO1 and P4, **b**), and *B. subtilis* strain ATCC6051 (**c**) were tested. Data are expressed as mean ± SEM of a single experiment (n = 6) that is representative of 3 independent experiments. Differences were analyzed using the unpaired Student’s t-test. Significance was shown as *P < 0.05, **P < 0.01.
