# Supplementary material for: Short chain fatty acids produced by Cutibacterium acnes inhibit biofilm formation by Staphylococcus epidermidis
Source: Sci Rep. 2020 Dec 4;10:21237. doi: 10.1038/s41598-020-77790-9 (PMC7718897; doi:10.1038/s41598-020-77790-9)

Supplementary Figures

Short chain fatty acids produced by *Cutibacterium acnes* inhibit biofilm formation by *Staphylococcus epidermidis*

Authors:
Kouki Nakamura[1], Alan M. O'Neill[1], Michael R. Williams[1], Laura Cau[1,2], Teruaki Nakatsuji[1], Alexander R. Horswill[3*], Richard L. Gallo[1]

Affiliations:
1 Department of Dermatology, University of California San Diego, La Jolla, California, USA.
2 SILAB, R&D Department, Brive, France
3 Department of Immunology and Microbiology, University of Colorado School of Medicine, Aurora, USA

Supplementary Figure 1

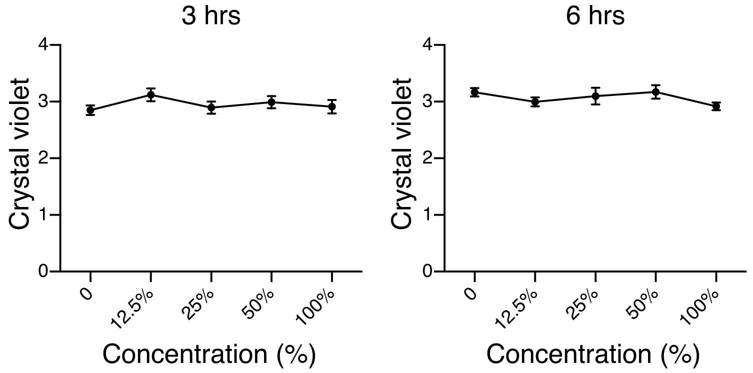

Supplementary Figure 2

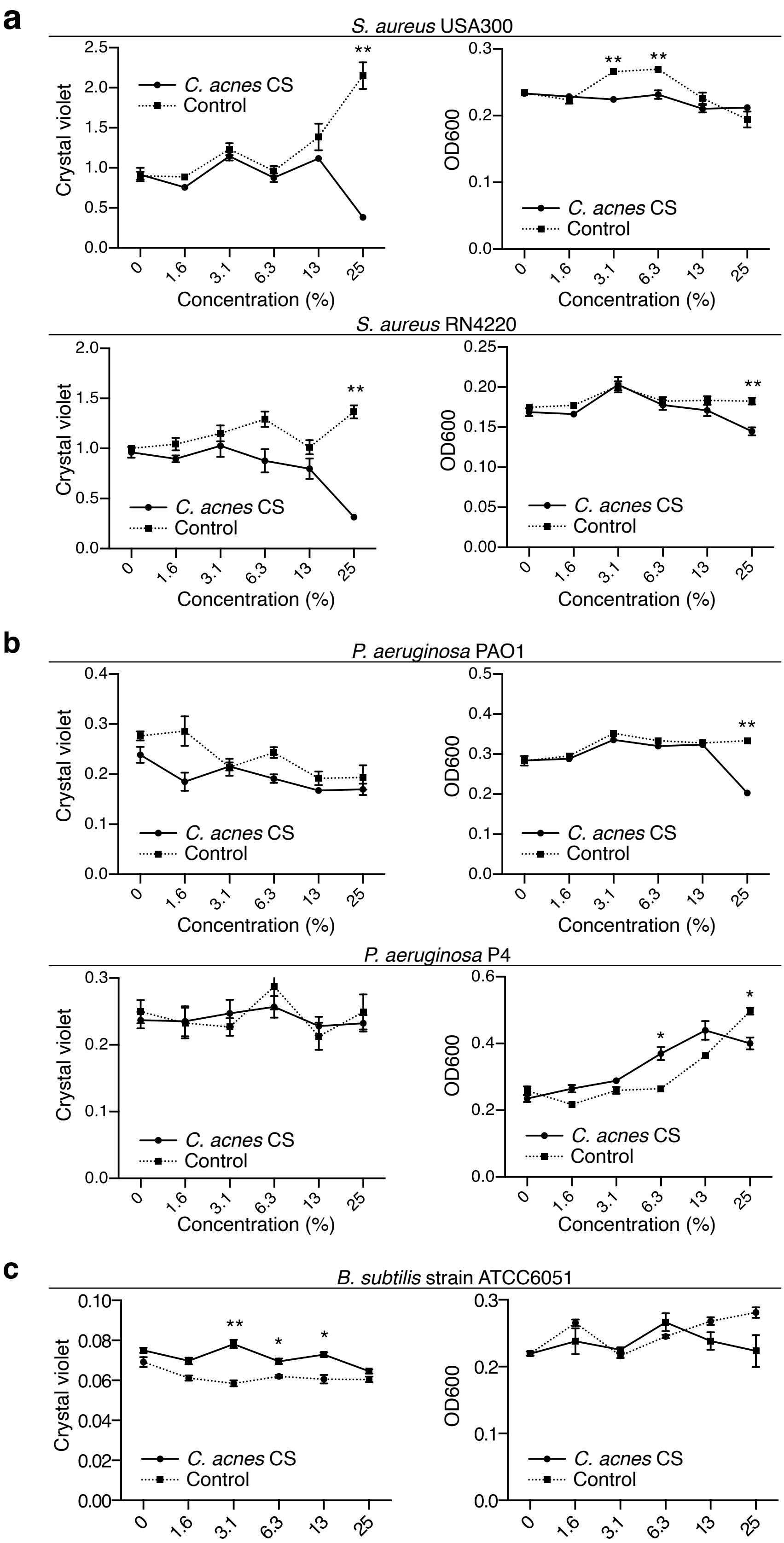

Supplement: Supplementary file 2 — Supplementary figures. [file 41598_2020_77790_MOESM2_ESM.pdf]
